# To troll or not to troll: Young adults’ anti-social behaviour on social media

**DOI:** 10.1371/journal.pone.0284374

**Published:** 2023-05-24

**Authors:** Felipe Bonow Soares, Anatoliy Gruzd, Jenna Jacobson, Jaigris Hodson

**Affiliations:** 1 London College of Communication, University of the Arts London, London, United Kingdom; 2 Ted Rogers School of Management, Toronto Metropolitan University, Toronto, Canada; 3 College of Interdisciplinary Studies, Royal Roads University, Victoria, Canada; University of Rome La Sapienza: Universita degli Studi di Roma La Sapienza, ITALY

## Abstract

**Background:**

Online anti-social behaviour is on the rise, reducing the perceived benefits of social media in society and causing a number of negative outcomes. This research focuses on the factors associated with young adults being perpetrators of anti-social behaviour when using social media.

**Method:**

Based on an online survey of university students in Canada (n = 359), we used PLS-SEM to create a model and test the associations between four factors (online disinhibition, motivations for cyber-aggression, self-esteem, and empathy) and the likelihood of being a perpetrator of online anti-social behaviour.

**Results:**

The model shows positive associations between two appetitive motives for cyber-aggression (namely recreation and reward) and being a perpetrator. This finding indicates that young adults engage in online anti-social behaviour for fun and social approval. The model also shows a negative association between cognitive empathy and being a perpetrator, which indicates that perpetrators may be engaging in online anti-social behaviour because they do not understand how their targets feel.

## Introduction

Anti-social behaviour on social media, such as harassment and bullying, is on the rise [1]. This trend has intensified since the beginning of the COVID-19 pandemic in 2020, when much social communication moved to online spaces [2–4]. Online anti-social behaviour can lead to several negative outcomes, such as decreasing an individual’s satisfaction with technologies and being online in general [5] to causing mental and emotional stress in victims [6]. Consequently, those at the receiving end of online anti-social behaviour (such as people who experience online harassment) may adopt coping strategies that can further isolate them [7].

In this study, we use the term “online anti-social behaviour” to encompass a range of harmful acts, including trolling (the intentional provocation of others through inflammatory online comments), bullying (aggressive behavior towards an individual or group), and harassment (offensive or abusive conduct directed at others) that have a negative impact, causing harm or distress to individuals or communities [8–10]. While bullying and harassment are related concepts, bullying is often defined as repeated aggressive behavior, typically by someone who perceives themselves to have more power over someone else [11]. Harassment, on the other hand, is a broader concept that includes any unwanted, offensive, or abusive conduct towards others.

While many studies on anti-social behaviour have focused on children and adolescents [12–16, for example], there is limited research focusing on young adults. Importantly, young adults are more likely than any other age group to report experiencing online harassment [1] and other forms of anti-social behaviour, especially during the COVID-19 restrictions [4]. Young adults are also generally more active online, particularly in Canada [17]. As such, the research focuses on university students.

This research focuses on the perpetrators of anti-social behaviour on social media and asks: What factors are associated with young adults being perpetrators of anti-social behaviour when using social media? The contributions of this research are twofold. First, most previous research has examined the intrinsic and extrinsic characteristics of people targeted by perpetrators of anti-social behaviour [see 1, 5, 6, 18]. Consequently, there is less understanding of what motivates perpetrators. Second, among the studies that focused on perpetrators, many looked at one or a few factors associated with the perpetration of anti-social behaviour [19–23]. Building on the previous scholarship, this research identifies and evaluates a more comprehensive model to understand psychological, social, and technology-associated factors related to being a perpetrator of online anti-social behaviour. Specifically, the proposed model incorporates the following factors known in the literature, but not necessarily tested together: online disinhibition, motivations for cyber-aggression, self-esteem, and empathy.

## Literature review

While social media can provide rewarding social connections for many, it can also be a space where users face anti-social behaviour. A recent study identified that 41% of Americans have personally experienced some form of online harassment or abuse; people who experienced online anti-social behaviour cited they were potentially targeted because of their political views, gender, race, ethnicity, religion and sexual orientation [1].

Anti-social behaviour is not a phenomenon exclusive to the internet; psychologists have widely analyzed anti-social behaviour in other contexts for several years prior to the widespread adoption of the internet [10]. The increased use of online platforms has contributed to the exponential rise of online anti-social behaviour [24, 25], which has, consequently, reduced the perceived benefits and promise of social media in society [26]. Recently, the increasing reliance on online platforms due to the COVID-19 pandemic restrictions has also been linked to the rise of anti-social behaviour [3, 4], perhaps because people have been spending more time on social media [2].

Online anti-social behaviour has several negative outcomes. First, it can reduce online participation, which is particularly impactful for minorities and marginalized communities. Lumsden and Harmer [27] identified that online anti-social behaviour is another avenue of disenfranchisement and discrimination for equity-deserving and marginalized communities, impacting their status, legitimation, and participation in online spaces. Second, previous research has shown that the effect of anti-social behaviour goes beyond the targets and also includes bystanders. Duggan [6] reported that 27% of Americans decided not to share something online after witnessing the abuse and harassment of others. Together, these negative effects of online anti-social behaviour can reduce the diversity of voices on social media and make people uncomfortable going online [28]. Third, online anti-social behaviour can have profound effects on individual’s emotional feelings, their reputation and personal safety [6].

While the effects of anti-social behaviour have been well documented, previous research is less clear on what makes someone engage in such behaviour towards another person online. To explain the prevalence of anti-social behaviour on social media and in public discourse, Hannan [29] revisited Neil Postman’s [30] theory about how the entertainment frame, which identifies the need for all information to be entertaining, has influenced public discourse. Focusing on television broadcasts in the last century, Postman [30] warned that the entertainment frame has seeped into education, journalism, and politics, which has changed how people interact with one another and society. In a society driven by an entertainment frame, individuals begin to expect all interactions to be entertaining, which influences behaviour and the boundaries of what communication is deemed acceptable. While Postman was writing about television, his theoretical lens has been effectively employed to understand social media [29]. Hannan [29] argued that, like television turned public discourse into “show business” the preeminence of online platforms has turned the online public sphere into a sort of “high school". Trolling on social media has become mainstream as a new genre of public speech, which shapes the discourse and the practices of politicians, public figures, and citizens.

To understand how the entertainment frame relates to a person’s likelihood to engage in online anti-social network, we developed a conceptual framework. The following section describes our conceptual framework which seeks to explain what makes someone engage in anti-social behaviour on social media. Specifically, we describe the factors and formulate a model of the drivers of the perpetration of online anti-social behaviour.

## Conceptual framework and research hypotheses

### Cyber-aggression

Since the goal of this research is to identify factors associated with being a perpetrator of anti-social behaviour on social media, Shapka’s and Maghsoudi’s [31] concept of cyber-aggression is applied. Instead of employing a binary classification and directly asking participants whether they consider themselves to be perpetrators or victims, the main dependent variable is the cyber-aggression construct. This construct assesses the level of people’s engagement in behaviour frequently associated with being a perpetrator, such as making hurtful comments about somebody’s race, ethnicity or sexual orientation, purposely excluding a certain person or group of people, and posting embarrassing photos or videos of someone else.

### Online disinhibition

Online disinhibition refers to the phenomenon when people say or do something online that they would not normally do in a face-to-face setting [32]. Suler [32] attributes this effect to six factors: [1] dissociative anonymity, as it is harder to determine who online people are; [2] invisibility, as people often cannot see each other online; [3] asynchronicity, as online communication does not require the sender and receiver to be co-present online for messages to be sent; [4] solipsistic introjection, as people tend to assign voices and other visual elements to whom they interact with due to the absence of face-to-face cues; [5] dissociative imagination, as some people can imagine separate dimensions from the real world when interacting online; and [6] minimization of status and authority, as people may perceive more of a peer-relationship as everyone “starts off on a level playing field” (p. 324) and therefore may be more willing to misbehave. Benign disinhibition refers to the effect when these factors motivate people to engage in positive interactions online. On the other hand, toxic disinhibition refers to when these factors motivate people to propagate hate and violence [32].

This study focuses on the association between online disinhibition and perpetration of online anti-social behaviour, as online disinhibition is linked to a higher likelihood of sharing harmful content [33]. Research suggests that use of social media enhances online disinhibition leading to anti-social behaviour [9]. Research has identified a positive association between online disinhibition and being a perpetrator of cyber-aggression [33–35]. In particular, Udris [35] separately analyzed the two dimensions of online disinhibition (i.e., benign disinhibition, and toxic disinhibition) and found that both positively predicted being a perpetrator. Wachs et al. [36] and Wachs and Wright [37] similarly found a positive association between the toxic dimension of online disinhibition and online hate. Building on this work, we propose the following hypothesis:

**H1. Online disinhibition is positively associated with being a perpetrator of cyber-aggression**. (Benign and toxic disinhibition are tested separately.)

### Motivations for cyber-aggression

Runions et al. [38] proposed a model to explore aggression motives based on the Quadripartite Violence Typology. This typology explores two dimensions: motivational valence and self-control. The motivational valence is aversive when the aggressive action of an individual is the reaction to violence or provocation. The motivational valence is appetitive when the motivation for one’s aggressive behaviour is to seek an exciting experience or some kind of reward. In summary, while aversive motivational valence is reactive, appetitive motivational valence is proactive. The self-control of aggressive actions might be impulsive or controlled depending on the deliberation and how it was planned. Based on the combination of the two dimensions, there are four distinct motivations for cyber-aggression: impulsive-aversive (Rage), controlled-aversive (Revenge), controlled-appetitive (Reward), and impulsive-appetitive (Recreation) [38].

Runions et al. [38] identified that all four motivations for cyber-aggression (i.e., Rage, Revenge, Reward, and Recreation) predicted being a cyber-aggression perpetrator. In terms of specific domains and different anti-social behaviours, Gudjonsson and Sigurdsson [20] found that excitement (Recreation) was a commonly endorsed motive for offending others. König et al. [23] found that victims of traditional bullying that engaged in cyberbullying tend to do it for revenge. Similarly, Fluck [14] identified that bullies indicate that their reason for engaging in cyber-aggression was mostly revenge, but also sadism attributed to fun experiences (Recreation) was mentioned for some bullies. Sadism was also found to be associated with online trolling, which indicates that trolls engage in anti-social behaviour for fun and enjoyment [39]. Thus, we expect that:

**H2. The motivations for cyber-aggression are positively associated with being a perpetrator of cyber-aggression**. (Each of the four motivations for cyber-aggression are tested separately).

### Self-esteem

Self-esteem refers to the perception one has towards the self [40, 41]. Self-esteem is usually viewed as a two dimensional construct: self-confidence and self-deprecation. Self-confidence refers to the positive attitudes towards the self. Self-deprecation focuses on negative perceptions towards the self. It is important to analyze the influence of self-esteem on cyber-aggression because self-esteem has been traditionally associated with offline anti-social behaviour, such as bullying [40]. Among research that explored the association between self-esteem and cyber-aggression, Rodríguez-Hidalgo et al. [15] found that self-deprecation was positively associated with being a perpetrator, but found nonsignificant associations between self-confidence and being a perpetrator. Other studies combined self-confidence and self-deprecation into a single construct of self-esteem (reverse-scoring items related to self-deprecation) and identified that lower levels of self-esteem lead to a higher likelihood of being a cyber-aggression perpetrator [40, 42]. Aligned with the prior work, we hypothesize that:

**H3. Self-esteem is negatively associated with being a perpetrator of cyber-aggression**. (Self-confidence and self-deprecation are assessed separately).

### Empathy

Empathy refers to the ability to experience and comprehend other people’s emotions and consists of two dimensions: the affective dimension (i.e., how one experiences the emotions of others) and the cognitive dimension (i.e., the capacity to comprehend the emotions of others) [43]. Empathy is relevant to understanding the motivations of anti-social behaviour because the capacity to experience and understand the emotions of others often leads to positive social interactions, such as helping others and sharing positive emotions and thoughts [12, 21]. In contrast, a lack of empathy may lead to negative social interactions.

Ang and Goh [12] found that both cognitive and affective empathy negatively predicted being a perpetrator of cyber-aggression. Jolliffe and Farrington [21] analyzed the influence of empathy in bullying among adolescents and found mixed results: both cognitive and affective empathy were negatively associated with bullying among boys, and only affective empathy was negatively associated with bullying among girls (the authors note that the low numbers of girls involved in bullying could have prevented cognitive empathy from reaching statistical significance). Casas et al. [44] analyzed empathy as a unidimensional construct (combining both cognitive and affective empathy) and found that low empathy leads to higher cyber-aggression perpetration. Other studies using adapted various scales to measure empathy found similar results [15, 22, 45].

In a systematic review, van Noorden et al. [16] identified that: (1) most studies reported a negative association between cognitive empathy and being a cyber-aggression perpetrator (although a few studies did not find any significant association or found a positive association), and (2) most studies reported a negative association between affective empathy and being a cyber-aggression perpetrator (with a few studies finding no association). Thus, we propose the following hypotheses:

**H4. Empathy is negatively associated with being a perpetrator of cyber-aggression**. (Cognitive and affective empathy are assessed separately).

Table 1 provides a summary of the research hypotheses. To identify factors associated with perpetration of anti-social behaviour, the scales included in the model have specific dimensions that can provide more granular results. Therefore, the model includes detailed scales to analyze how each factor is associated with being a perpetrator of online anti-social behaviour.

**Table 1:** Research hypotheses.

Factors	Hypotheses
**Online disinhibition**	H1a. Benign online disinhibition is positively associated with being a perpetrator of cyber-aggression.
	H1b. Toxic online disinhibition is positively associated with being a perpetrator of cyber-aggression.
**Motives for cyber-aggression**	H2a. Rage is positively associated with being a perpetrator of cyber-aggression.
	H2b. Revenge is positively associated with being a perpetrator of cyber-aggression.
	H2c. Reward is positively associated with being a perpetrator of cyber-aggression.
	H2d. Recreation is positively associated with being a perpetrator of cyber-aggression.
**Self-esteem**	H3a. Self-deprecation is positively associated with being a perpetrator of cyber-aggression.
	H3b. Self-confidence is negatively associated with being a perpetrator of cyber-aggression.
**Empathy**	H4a. Cognitive empathy is negatively associated with being a perpetrator of cyber-aggression.
	H4b. Affective empathy is negatively associated with being a perpetrator of cyber-aggression.

## Methods

Prior to data collection, the study received approval from the Research Ethics Boards at both Toronto Metropolitan University and Royal Roads University (at the time of the study, the authors were affiliated with one of these institutions). Undergraduate students at Toronto Metropolitan University who signed up for the Student Research Participant Pool were invited to voluntarily participate in an online survey. The Student Research Participant Pool invites students to voluntarily participate in scholarly research and receive extra course credit that can be applied to specific courses.

Before taking the survey, participants were required to review and agree to the informed consent form before starting the survey which was hosted on Qualtrics, an online platform. Students were given the opportunity to review and save the consent form on their own devices. They were also able to withdraw from the survey at any time by simply closing their browser. In such cases, their data was not used in the study. As this was an online survey, students had the flexibility to complete it at their own pace and from any location of their choosing.

In total, 557 students participated in the survey between March 9 and April 18, 2022. The survey dataset was cleaned and the data was completely anonymized. A two-step disqualification process was used to assure the high quality of the data. First, an attention check question was employed to identify participants who were not carefully reading the questions, which resulted in the removal of 182 responses who answered the question incorrectly. Second, responses from participants who completed the survey in less than 5 minutes, which indicates that they did not carefully read the questions (n = 16), were removed. We did not exclude responses that took longer than expected because some students may have opened the survey page but completed it at a later time. After data cleaning, the final dataset consisted of 359 participants. On average, respondents completed the survey in 25 minutes, and the median completion time was 13 minutes, which was aligned with the anticipated completion time in the piloted survey. The final dataset is available at doi.org/10.6084/m9.figshare.22185994.

Partial Least Squares Structural Equation Modeling (PLS-SEM) was used to analyze the data. PLS-SEM is a non-parametric approach that can handle complex models and can be used to test relationships between multiple independent and dependent variables simultaneously [46, 47]. This method has been widely used in several fields, such as business, political communication, and psychology [48, 49], and more recently internet studies [50–52]. SmartPLS v. 3.3.9 software was used to analyze the association between the constructs below.

### Measurement scales

The scales used in the online survey have been tested and validated by previous research. All constructs were measured using a 5-point Likert scale ranging from “strongly disagree” to “strongly agree,” except for the measurement of being a perpetrator of online anti-social behaviour, which was measured using a 5-point Likert scale ranging from “never” to “always.” S1 Appendix outlines the constructs and scales used in the research. Based on the previous applications of these scales, all were modeled as reflective constructs in the PLS-SEM analysis.

Cyber-aggression was measured using the Cyber-aggression and Cyber-victimization Scale [31]. While this scale has two components: cyber-aggression and cyber-victimization, only the former was used in our research (CAVP) due to the focus on perpetrators of anti-social behaviour. The scale included twelve indicators with statements about how individuals behave toward others online, such as “posted or re-posted something embarrassing or mean about another person.” This scale is particularly useful because it focuses on cyber-aggressive behaviour overall (i.e., specific acts associated with cyber-aggression). This scale overcomes a limitation of previous scales that focused on specific online platforms (e.g., Facebook) or modes of communicating (e.g., computers or cellphones) [31].

The Online Disinhibition Scale [35] was used to measure benign disinhibition (BOD) and toxic disinhibition (TOD). Benign disinhibition was measured by seven indicators and toxic disinhibition was measured by four indicators.

To measure the four motivations for cyber-aggression, an adapted version of the Cyber-Aggression Typology Questionnaire [25] was used. In Antipina et al.’s [13] adaptation, each motive (i.e., Rage, Revenge, Reward, and Recreation) was measured by five indicators.

To evaluate the levels of empathy of respondents, the Rosenberg’s Self-Esteem Scale [41] was used whereby the two dimensions of self-esteem were separately explored. Self-confidence (RSEC) and self-deprecation (RSED) were each measured by five indicators.

The Basic Empathy Scale [43] was used to explore cognitive empathy (BCE) and affective empathy (BAE). Cognitive empathy was measured by nine indicators and affective empathy was measured by eleven indicators.

Table 2 provides descriptive data of the constructs in our dataset.

**Table 2:** Descriptive data about the factors included in the model.

Construct	N	Mean	Std. Deviation
**CAVP**	359	1.20	0.43
**BOD**	359	3.08	0.81
**TOD**	359	1.73	0.78
**Rage**	359	1.66	0.84
**Revenge**	359	1.52	0.75
**Reward**	359	1.30	0.61
**Recreation**	359	1.44	0.72
**RSEC**	359	3.87	0.80
**RSED**	359	2.83	0.98
**BAE**	359	3.60	0.68
**BCE**	359	4.03	0.53

## Constructs and model assessments

Current PLS-SEM guidelines were followed to assess the reliability of the constructs, the validity of the model, and to report the results [47, 53]. The following procedures for the constructs and model assessments were used: internal consistency, discriminant validity, collinearity between indicators, and significance and relevance of the structural model.

We identified issues of internal consistency in five constructs: Affective Empathy (BAE), Cognitive Empathy (BCE), Benign Online Disinhibition (BOD), and Self-Deprecation (RSED). Additionally, we identified indicators with low outer loadings for Toxic Online Disinhibition (TOC). To solve these issues, we removed indicators with loadings below 0.6. Although the ideal threshold is 0.7, a threshold of 0.6 is acceptable for exploratory research [53]. We decided to use the 0.6 threshold for outer loadings because the more conservative 0.7 threshold would cause the Cronbach’s alpha for BOD to go below the minimum acceptable value of 0.6. After excluding six BAE indicators, five BCE indicators, four BOD indicators, two TOD indicators, and two RSED indicators, values of composite reliability were well above the minimum of 0.6, and values of Average Variance Extracted (AVE) were above the minimum of 0.5 for all constructions. Cronbach’s alpha values were above the ideal 0.7 for most constructs, except for BOD and BCE, which were above the minimum acceptable of 0.6. In total, we removed 26% of the indicators, which is within acceptable limits for exploratory research [54]. We have verified that the majority of constructs (excluding toxic online disinhibition) were assessed using at least three items, which is considered ideal for statistical identification of the construct [54]. Table 3 details the internal consistency values, while Table 4 displays the loadings of the indicators.

**Table 3:** Internal consistency.

	Cronbach’s Alpha	rho_A	Composite Reliability	Average Variance Extracted (AVE)
**BAE**	0.834	0.845	0.882	0.601
**BCE**	0.677	0.679	0.8	0.502
**BOD**	0.618	0.684	0.786	0.555
**CAV-P**	0.939	0.941	0.947	0.601
**RSEC**	0.855	0.898	0.893	0.626
**RSED**	0.803	1.333	0.857	0.671
**Rage&Rev**	0.92	0.923	0.933	0.583
**Recreation**	0.878	0.881	0.911	0.671
**Reward**	0.875	0.876	0.909	0.668
**TOD**	0.788	0.788	0.904	0.825

**Table 4:** Loadings of the indicators.

	BAE	BCE	BOD	CAV-P	RSEC	RSED	Rage&Rev	Recreation	Reward	TOD
**BES_A1**	0.814									
**BES_A13**	0.76									
**BES_A18**	0.831									
**BES_A7**	0.713									
**BES_A8**	0.752									
**BES_C14**		0.625								
**BES_C19**		0.734								
**BES_C20**		0.692								
**BES_C6**		0.775								
**Benign2**			0.746							
**Benign3**			0.614							
**Benign6**			0.854							
**CAV-P_1**				0.742						
**CAV-P_10**				0.831						
**CAV-P_11**				0.833						
**CAV-P_12**				0.807						
**CAV-P_2**				0.745						
**CAV-P_3**				0.749						
**CAV-P_4**				0.752						
**CAV-P_5**				0.668						
**CAV-P_6**				0.784						
**CAV-P_7**				0.724						
**CAV-P_8**				0.831						
**CAV-P_9**				0.821						
**RSES_SC1**					0.801					
**RSES_SC10**					0.841					
**RSES_SC3**					0.774					
**RSES_SC4**					0.707					
**RSES_SC7**					0.826					
**RSES_SD5**						0.715				
**RSES_SD6**						0.761				
**RSES_SD9**						0.96				
**Rage3**							0.831			
**Rage5**							0.768			
**Rage7**							0.726			
**Rage8**							0.715			
**Rage9**							0.763			
**Recreation1**								0.826		
**Recreation2**								0.812		
**Recreation3**								0.85		
**Recreation4**								0.832		
**Recreation5**								0.775		
**Revenge2**							0.784			
**Revenge3**							0.801			
**Revenge4**							0.727			
**Revenge5**							0.756			
**Revenge6**							0.76			
**Reward1**									0.853	
**Reward2**									0.798	
**Reward3**									0.824	
**Reward4**									0.841	
**Reward5**									0.768	
**Toxic1**										0.908
**Toxic4**										0.909

We also identified one discriminant validity issue. The HTMT correlation between Rage and Revenge was above 0.95, which suggests that both constructs were not empirically distinct from each other in the model. Therefore, we decided to combine the two constructs into one, since both focus on aversive motives for cyber-aggression [25, 38]. This approach is aligned with prior research on the motivational valence of cyber-aggression [55]. After creating a single construct for aversive motives (Rage and Revenge), no other discriminant validity issues were identified (see Table 5). There were no collinearity issues in the data, as VIF values were below 0.5 for all indicators.

**Table 5:** Discriminant validity—heterotrait-monotrait ratio of correlations (HTMT).

	**BAE**	**BCE**	**BOD**	**CAV-P**	**RSEC**	**RSED**	**Rage&Rev**	**Recreation**	**Reward**	**TOD**
**BAE**										
**BCE**	0.565									
**BOD**	0.134	0.218								
**CAV-P**	0.284	0.311	0.137							
**RSEC**	0.075	0.359	0.18	0.07						
**RSED**	0.096	0.31	0.34	0.11	0.696					
**Rage&Rev**	0.217	0.217	0.465	0.471	0.072	0.265				
**Recreation**	0.311	0.281	0.368	0.597	0.071	0.138	0.746			
**Reward**	0.333	0.273	0.383	0.595	0.069	0.142	0.837	0.84		
**TOD**	0.359	0.274	0.501	0.477	0.058	0.079	0.601	0.84	0.694	

Values of path (β) coefficients, F^2^, and R^2^ were considered to measure the relevance of the model, while bootstrapping was used to test the significance of the associations between constructs.

## Results

**Fig 1:**
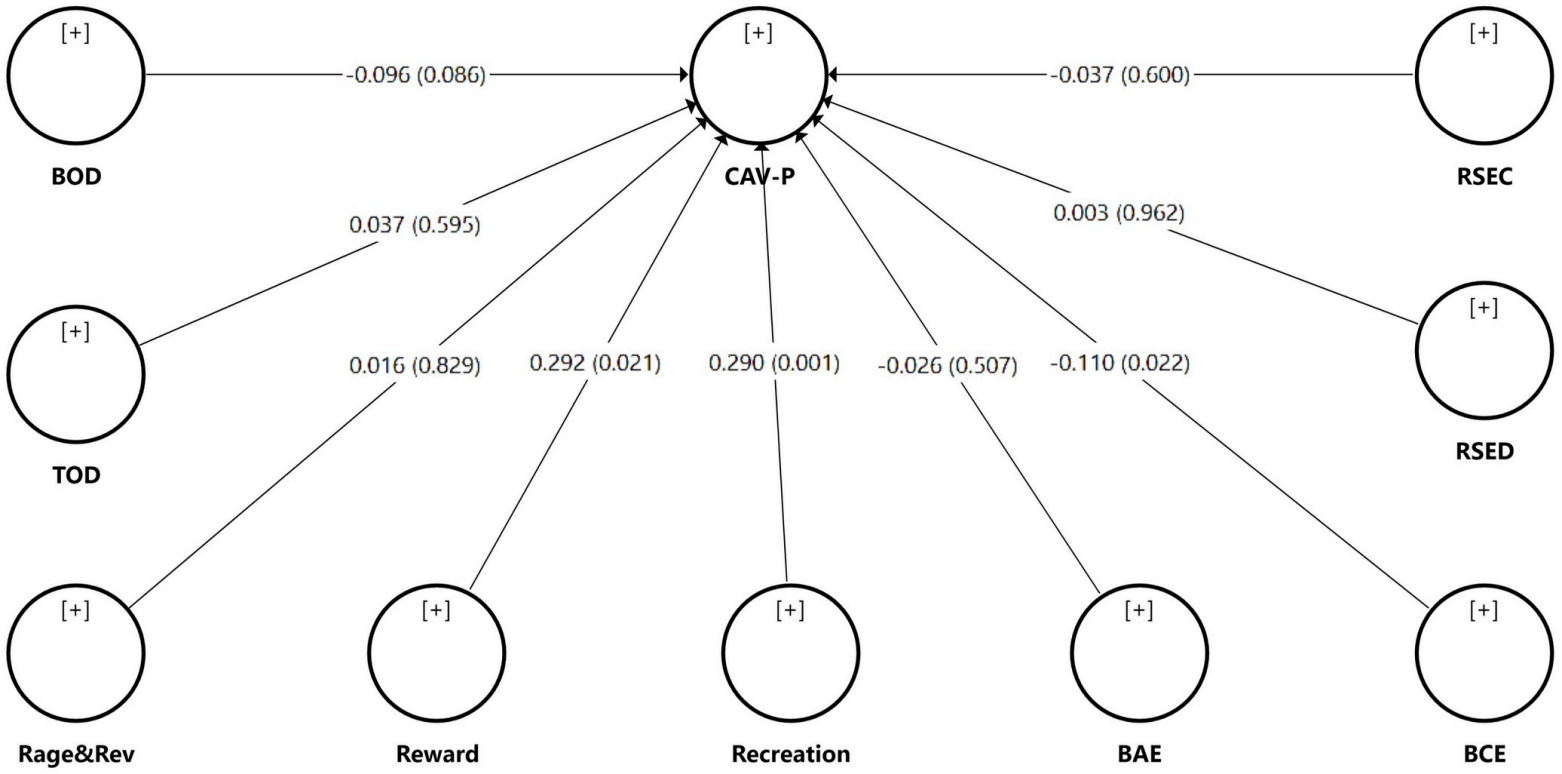
Results of the structural model assessment. Note: For each connection between constructs, β coefficients and *p* values (in brackets) are displayed.

The analysis of the model (see Fig 1) shows a moderate positive and significant association between reward and being a perpetrator (β = 0.292), and between recreation and being a perpetrator (β = 0.290), which supports H2c and H2d. The analysis also indicates a weak but significant negative association between cognitive empathy and being a perpetrator (β = -0.110), which supports H4a. No other construct had a significant association with being a perpetrator of cyber-aggression. Table 6 provides detailed information about which hypotheses were supported by the results. The assessment of effect sizes shows small effect size of reward and recreation on being a perpetrator (both f^2^ = 0.043), and near negligible effect size of cognitive empathy on being a perpetrator (f^2^ = 0.014).

**Table 6:** Results of the tested hypotheses.

Factors	Hypotheses	Results
**Online disinhibition**	H1a. Benign online disinhibition is positively associated with being a perpetrator of cyber-aggression.	Not supported
	H1b. Toxic online disinhibition is positively associated with being a perpetrator of cyber-aggression.	Not supported
**Motives for cyber-aggression**	H2a. Rage is positively associated with being a perpetrator of cyber-aggression.	Not supported
	H2b. Revenge is positively associated with being a perpetrator of cyber-aggression.	Not supported
	H2c. Reward is positively associated with being a perpetrator of cyber-aggression.	**Supported**
	H2d. Recreation is positively associated with being a perpetrator of cyber-aggression.	**Supported**
**Self-esteem**	H3a. Self-deprecation is positively associated with being a perpetrator of cyber-aggression.	Not supported
	H3b. Self-confidence is negatively associated with being a perpetrator of cyber-aggression.	Not supported
**Empathy**	H4a. Cognitive empathy is negatively associated with being a perpetrator of cyber-aggression.	**Supported**
	H4b. Affective empathy is negatively associated with being a perpetrator of cyber-aggression.	Not supported

In terms of model assessment and explanatory power, the model shows a moderate predictive power (adj. R^2^ = 0.352) and the SRMR indicates a good model fit (0.057 for the saturated model and for the estimated model). The blindfolding procedure with a distance omission of 7 returns positive values of Q^2^ = 0.202, which confirms the predictive relevance of the model.

## Discussion

In the model, the findings suggest that recreation and reward are two important constructs to understand the perpetration of online anti-social behaviour. In the context of our research, this indicates that appetitive motives for anti-social behaviour (i.e., when the aggression is proactive) are more important than aversive motives (i.e., rage and revenge), in which the aggression is a reaction to another situation. Our findings are consistent with studies that focused on online trolls [39] and young offenders on probation [20], and contrary to studies that focused on bullying and cyber-bullying [14, 23]. While online trolls and young people on probation indicate that they engage in online anti-social behaviour for fun, enjoyment, and excitement (related to appetitive motives), bullies and cyber-bullies tend to indicate revenge as their main reason. Therefore, young people in our sample engaging in anti-social behaviour might be seeking excitement and aiming to obtain positive emotions or social status [25, 38]. In this sense, self-control, which distinguishes recreation (impulsive) from reward (controlled) does not seem to play a significant role in the likelihood of young people engaging in anti-social behaviour.

A previous study that explored the role of different motivations in online and offline aggression [19] found that recreation was more prevalent in online environments, which is aligned with our findings. Graf et al. [19] suggest that recreation may be prevalent online because this motivation is generally associated with less interpersonal motives. On the other hand, Graf et al. [19] identified that reward was more prevalent in the offline context, especially because this motivation is generally associated with social dynamics such as group affiliation and power relations [18, 19, 56]. Therefore, perpetrators seeking rewards often prefer offline environments because they have more control over the bystanders and how they will shape social structure as a consequence of their acts [19]. Facing the COVID-19 pandemic, young people have been spending more time online, reducing the access to in-person activities in which they could have engaged in anti-social behaviour for reward purposes. This could explain why reward was identified as a prevalent reason for young people to engage in online anti-social behaviour; they had to adapt how they interact with others in a context that was heavily dependent on online platforms for social interactions.

The data generally supports both Postman’s [30] theory of the entertainment frame and how it was later modernized by Hannan [29]. Specifically, we found that university students engage in anti-social behaviour both for fun (i.e., recreation) and social approval (i.e., reward). Perpetrators of anti-social behaviour on social media are doing so because it is entertaining. While recreation is strongly associated with the original theory and the centrality of entertainment in public discourse, reward emerges as particularly important when the theory was revisited by Hannan [29] to account for how social media affected the public discourse, making trolling a central feature of social interactions that emulate a high school setting.

In addition to reward and recreation, the model shows that cognitive empathy is also a factor associated with the perpetration of online anti-social behavior. Those with lower cognitive empathy, indicating a lower capacity to comprehend the emotions of others, are more likely to engage in such behavior. This suggests that perpetrators may be engaging in online anti-social behavior because they do not fully understand how their targets feel. Based on this finding, one potential strategy for reducing the prevalence of online anti-social behavior is to implement psychological interventions that highlight the negative effects of the behavior on the targets.

Interestingly, other factors showed nonsignificant associations with cyber-aggression perpetration. The fact that both benign and toxic online disinhibition had nonsignificant associations with perpetration indicates that characteristics of online platforms (e.g., anonymity and asynchronicity) and perceptions of social norms in online interactions (e.g., minimization of status and authority) do not play a significant role in online anti-social behaviour among university students. Although studies and reports indicated that the prevalence of online anti-social acts (such as online harassment and cyber-bullying) increased during the pandemic [2–4], our results indicate that the spike in online anti-social behaviour is less about online disinhibition and more about how most social interactions moved to the online environment. Instead of being a consequence of the online environment, anti-social behaviour is more likely motivated by the need for social approval, group bonding, fun and excitement (as indicated by the positive associations with reward and recreation).

There were no significant associations between any dimensions of self-esteem (i.e., self-confidence and self-deprecation) and being a perpetrator. Therefore, the results do not support findings from previous studies that identified an association between self-esteem and perpetration [15, 40, 42]. Our data suggests that one’s perception towards the self is not a key factor of being a perpetrator, at least not among the studied population.

In summary, this study provides evidence on why young adults, particularly university students, engage in anti-social behavior. By highlighting the association between engagement in anti-social behavior and social factors such as enjoyment and social approval, our study presents a direction for future research to further analyzehow social elements play a role in anti-social behavior. While engagement in various forms of anti-social behavior is frequently linked to psychological traits, we found cognitive empathy to be the only significant factor among our study participants. In particular, a lower ability to understand how targets feel may be fueling the desire for fun and social approval without regard for the consequences. Future studies can further explore the relationship between these constructs.

## Conclusion

The research sought to identify the factors associated with the perpetration of anti-social behaviour. We developed a model to account for the role of online disinhibition, motivations for cyber-aggression, self-esteem, and empathy in the perpetration of online anti-social behaviour.

The findings suggest that three factors are associated with the perpetration of online anti-social behaviour: recreation, reward and cognitive empathy. Both recreation and reward are appetitive motives for anti-social behaviour, which suggests that young people engage in online anti-social behaviour for fun, excitement, and social approval. Cognitive empathy was negatively associated with the perpetration of online anti-social behaviour, which suggests that perpetrators have lower capacity to comprehend the emotions of others. Perpetrators have a lower understanding of how their targets might feel and this could partly explain why they engage in online anti-social behaviour.

Other factors showed nonsignificant associations with perpetration. Interestingly, both benign and toxic disinhibition had nonsignificant associations with perpetration, which indicates that the prevalence of online anti-social behaviour is less about the nature of the medium (e.g., anonymity, asynchronicity) and more about individuals involved.

Building on the results, there are two potential strategies in mitigating anti-social behaviour. First, related to our findings that perpetrators are more likely to be motivated by recreation and reward and have lower cognitive empathy, we refer to earlier work by Jolliffe and Farrington [21] who found that making people think about their actions increases their awareness and builds empathy towards the target. In this regard, strategies such as Twitter’s intervention to add friction to make people reconsider when posting potentially offensive content [57] might be a strategy to reduce anti-social behaviour on social media. These types of strategies may be useful both in terms of making people think about their targets and potentially understand how they might feel (cognitive empathy), and reducing impulsive anti-social acts (recreation). For example, a recent survey of Twitter users who had posts removed by the platform found that less than 2% of them posted something to intentionally hurt someone [58].

Second, while outside the scope of the current study, Kim et al. [59] found that showing basic community guidelines to users can also encourage individuals to engage in healthier discussions, reducing the number of problematic content that was reported by others. This suggests that in addition to introducing some friction into online communication, platforms should endeavour to include more education in highlighting community rules and norms set by a given platform or an online community. This way, newcomers to the platform would learn what is and is not acceptable behaviour in a given community from the beginning. While this idea is not new, various communities on Reddit have already adopted this approach; most larger social media platforms tend to develop long, jargon-ridden guidelines of community norms, which are then buried in the fine print and are not seen or read by users [60]. Katsaros et al. [58] found that one in five users who violated Twitter’s rules has never read the platform’s guidelines on appropriate behaviour, and of those who have read the rules, over half of them were merely somewhat familiar or less familiar with them.

As with any empirical work, the research has several limitations that stimulate future research in this area. Since this study relies on a sample of undergraduate students from one urban university in Canada, our sample is only representative of this group of young adults. Future studies could expand the work by using different and/or larger samples, such as nationally representative samples of adults. The reliability of some scales were also below the expected threshold, an issue that was solved by following the current PLS-SEM procedures. Therefore, future studies can revalidate some of these scales by using larger and/or more diverse samples.

## Supporting information

S1 Appendix Constructs. (DOCX)
