# Supplementary material for: To troll or not to troll: Young adults’ anti-social behaviour on social media
Source: PLoS One. 2023 May 24;18(5):e0284374. doi: 10.1371/journal.pone.0284374 (PMC10208514; doi:10.1371/journal.pone.0284374)
Supplement: S1 Appendix — (DOCX) [file pone.0284374.s001.docx]

**S1 Appendix. Constructs**

**Cyber-aggression and Cyber-victimization Scale** (Shapka & Maghsoudi, 2017)

Please indicate how often YOU behaved as described below towards OTHERS on social media.

[Never, Rarely, Sometimes, Often, Always]

| Construct | Survey questions |
| --- | --- |
| Cyber-aggression | - Posted or re-posted something embarrassing or mean about another person - Sent or forwarded a hurtful message to someone - Posted or re-posted an embarrassing photo or video of someone that they did not want others to see - Posted a hurtful comment about an online photo or video of somebody else (for example, made fun of how they look) - Posted messages to purposely exclude a certain person or group of people - Posted or re-posted something private about another person that they did not want others to know - Spread rumours or gossip about someone - Made hurtful comments about somebody's race or ethnicity - Made hurtful comments about somebody's perceived sexual orientation - Made hurtful comments about somebody's perceived sexual behaviors (for example, called somebody a slut or a pervert) - Said something sexual to somebody else to embarrass them or to be mean - Sent sexual content (photos or jokes) to somebody else to embarrass them or to be mean |

**Online Disinhibition Scale** (Udris, 2014)

Please indicate the extent to which you agree with the following statements:

[Strongly disagree, Somewhat disagree, Neither agree nor disagree, Somewhat agree, Strongly agree]

| Construct | Survey questions |
| --- | --- |
| Benign disinhibition | - It is easier to connect with others via social media than talking in person - Social media is anonymous so it is easier for me to express my true feelings or thoughts - It is easier to post things on social media that would be hard to say in real life because you don’t see the other’s face - It is easier to communicate via social media because you can reply anytime you like - I have an image of the other person in my head when I read their social media post(s) - I feel like a different person when I use social media - I feel that on social media I can communicate on the same level with others who are older or have higher status |
| Toxic disinhibition | - I don’t mind writing insulting things about others on social media because it’s anonymous - It is easy to post insulting things on social media because there are no repercussions - There are no rules on social media therefore you can do whatever you want - Posting insulting content on social media is not bullying |

**Cyber-Aggression Typology Questionnaire** (Runions et al., 2017 - adapted by Antipina et al., 2019)

How much do you agree with the statements below?

[Strongly disagree, Somewhat disagree, Neither agree nor disagree, Somewhat agree, Strongly agree]

| Construct | Survey questions |
| --- | --- |
| Rage | - I use social media to get back at someone as soon as they post a hurtful message about me - If someone makes fun of me on the internet, I get frustrated and respond angrily online right away - If I see a message on social media that gets me angry, I react too quickly and then regret the way I responded - If someone tries to cyberbully me, I quickly lash back with something online - If someone says something online to hurt me, I post something back right away to get back at them |
| Revenge | - If someone tries to hurt me, I will use social media to get back at them in my own time - I get back at people who make fun of me on social media because their posts hurt more the more I think about them - I like using social media to plan my revenge when I feel angry at someone - If I need to get revenge on someone, I would rather strike back using social media where I can plan out how to do it - If I see a mean message about me on social media, it bothers me more and more when I think about it, and I try to get even |
| Reward | - If I don’t like someone, I use social media to turn others against them - Sometimes I’ll team up with my friends to bring someone down on social media - Sometimes I can be mean to people on social media to get what I want - When I don’t like a person, I use social media to make them feel like they do not belong in my group - I pretend to be someone else online to ruin somebody else’s friendships |
| Recreation | - I get carried away having fun online and others think I’m being a cyberbully or a troll - I make fun of people I don’t know on social media without thinking about whether they will see it or not - If I’m having fun and joking online, I don’t care if someone’s feelings get hurt - I repeatedly annoy people online because I think it’s funny - Joking on social media is so much fun that I don’t worry about whether someone might be bothered by what I say |

**Rosenberg's Self-Esteem Scale** (Rosenberg, 2015)

Below is a list of statements dealing with your general feelings about yourself. Please indicate how strongly you agree or disagree with each statement.

[Strongly disagree, Somewhat disagree, Neither agree nor disagree, Somewhat agree, Strongly agree]

| Construct | Survey questions |
| --- | --- |
| Self-confidence | - On the whole, I am satisfied with myself - I feel that I have a number of good qualities - I am able to do things as well as most other people - I feel that I'm a person of worth, at least on an equal plane with others - I take a positive attitude toward myself |
| Self-deprecation | - At times I think I am no good at all - I feel I do not have much to be proud of - I certainly feel useless at times - I wish I could have more respect for myself - All in all, I am inclined to feel that I am a failure |

**Basic Empathy Scale** (Jolliffe & Farrington, 2006)

Think about your ability to understand and share the feelings of another. For each statement below, please indicate your agreement or disagreement.

[Strongly disagree, Somewhat disagree, Neither agree nor disagree, Somewhat agree, Strongly agree]

| Construct | Survey questions |
| --- | --- |
| Affective empathy | - My friends’ emotions don’t affect me much - After being with a friend who is sad about something, I usually feel sad - I get frightened when I watch characters in a good scary movie - I get caught up in other people’s feelings easily - I don’t become sad when I see other people crying - Other people’s feeling don’t bother me at all - I often become sad when watching sad things on TV or in films - Seeing a person who has been angered has no effect on my feelings - I tend to feel scared when I am with friends who are afraid - I often get swept up in my friends’ feelings - My friend’s unhappiness doesn’t make me feel anything |
| Cognitive empathy | - I can understand my friend’s happiness when they do well at something - I find it hard to know when my friends are frightened - When someone is feeling ‘down’ I can usually understand how they feel - I can usually work out when my friends are scared - I can often understand how people are feeling even before they tell me - I can usually recognize when people are cheerful - I can usually realize quickly when a friend is angry - I am not usually aware of my friends’ feelings - I have trouble figuring out when my friends are happy |
